# Awareness and Use of Post-exposure Prophylaxis for HIV Prevention Among Men Who Have Sex With Men: A Systematic Review and Meta-Analysis

**DOI:** 10.3389/fmed.2021.783626

**Published:** 2022-01-10

**Authors:** Junyan Jin, Runsong Sun, Tingting Mu, Taiyi Jiang, Lili Dai, Hongyan Lu, Xianlong Ren, Jing Chen, Jingrong Ye, Lijun Sun, Hao Wu, Tong Zhang, Huachun Zou, Bin Su

**Affiliations:** ^1^Beijing Key Laboratory for HIV/AIDS Research, Sino-French Joint Laboratory for Research on Humoral Immune Response to HIV Infection, Clinical and Research Center for Infectious Diseases, Beijing Youan Hospital, Capital Medical University, Beijing, China; ^2^School of Sociology, Beijing Normal University, Beijing, China; ^3^Institute for AIDS/STD Control and Prevention, Beijing Center for Disease Prevention and Control, Beijing, China; ^4^School of Public Health (Shenzhen), Sun Yat-sen University, Shenzhen, China

**Keywords:** post-exposure prophylaxis, MSM, awareness, meta-analysis, HIV

## Abstract

**Background:** The use of post-exposure prophylaxis (PEP) is effective in reducing HIV risk, but it is underused by men who have sex with men (MSM) due to certain psychological and sociostructural factors. This article assessed the awareness and use of PEP among MSM in an effort to increase the visibility and uptake of PEP among at-risk populations.

**Methods:** We conducted a systematic literature search of the PubMed, Web of Science, PsycINFO, and Google Scholar electronic databases. Studies were screened for inclusion, and relevant data were abstracted, assessed for bias, and synthesized. Pooled effect estimates were calculated using random effects meta-analysis, meta-regression and subgroup analysis, and a qualitative review and risk of bias assessment were performed (PROSPERO, CRD42019123815).

**Results:** Twenty eligible studies involving 12,579 MSM were included in the meta-analysis. The pooled estimate of the proportions of MSM who were aware of PEP was modest at 59.9% (95% CI: 50.5~68.7) and that of MSM who previously used PEP was very low at 4.9% (95% CI: 2.4~9.8). PEP awareness showed no clear change over time, while PEP use significantly changed over time. Multiple factors affected awareness, including educational attainment, race/ethnicity, levels of HIV stigma, access to condoms, and so on. Many factors could potentially impede or facilitate the use of PEP, such as income, lack of PEP information, and partnership.

**Conclusion:** We observed that PEP is an underused HIV prevention strategy among MSM and that once MSM become aware of PEP, the majority are willing to use it if they are supported appropriately in terms of a range of individual, social, and structural barriers.

**Systematic Review Registration**: http://www.cdr.york.ac.uk/prospero, PROSPERO [CRD42019123815].

## Introduction

Gay men, bisexual men, and other men who have sex with men (MSM) are a critical population at risk for HIV/AIDS throughout the world (1). The HIV prevalence among MSM exceeds 10% in many regions, which is disproportionately high compared to the general population (2). In many highly developed countries where the overall HIV epidemic is in decline, there have been re-emergent epidemics among MSM (3). In low- and middle-income settings, the epidemic of HIV among MSM was expanding (4).

Chemoprophylaxis for HIV prevention, including pre- and post-exposure prophylaxis (PrEP/PEP), has emerged as an important component of HIV prevention efforts in recent years (5). PEP is a 4-week combination antiretroviral treatment beginning within 72 h of initial exposure or potential exposure to human body fluids possibly infected with HIV (6). PEP was initially used for the prevention of occupational HIV exposure, and recently, research hotspots have demonstrated its safety and feasibility in non-occupational incidents among high-risk populations such as MSM (7). However, PEP has been shown to be underused by those who experience non-occupational exposure (8–10).

Relatively low awareness and willingness to use PEP was prevalent among MSM. Some researchers found that most MSM (88.3%) in London had heard of PEP (11), but more studies showed that awareness of PEP was relatively low among that population (12–14), and a lower percentage (24%) of individuals at risk were aware of the proper timing of effective PEP treatment (15). One study showed that of the responders, 42.5% was aware of PEP, and 59.9% expressed interest in receiving PEP in the future, if required (16). A meta-analysis revealed that only 67.2% of MSM were willing to complete the full 28-day uptake of antiretroviral drugs prescribed for PEP (17). Another meta-analysis showed that the pooled PEP uptake rate among high-risk MSM was only 5.0% (14). Many factors may hinder PEP uptake among MSM, including experiences of racism, homophobia, and HIV stigma (18, 19), suggesting that PEP awareness and uptake may be influenced by certain psychological and sociostructural factors. Therefore, increased attention should be devoted to effectively increasing the use of PEP (20) and reducing HIV incidence among MSM (21).

The AIDS epidemic is hidden to some degree because of continued stigma, discrimination, and violence (22). To reduce new HIV infections, it is essential for medical and health services to expand to cover MSM as a key population. In an effort to understand how to raise the visibility and uptake of PEP within at-risk populations, we conducted a systematic review and meta-analysis that assessed PEP awareness and use among MSM, and we sought to examine factors associated with that.

## Methods

### Search Strategy

This article was preregistered in the International Prospective Register of Systematic Reviews (PROSPERO, http://www.cdr.york.ac.uk/prospero, CRD42019123815). The work was reported in accordance with the Preferred Reporting Items for Systematic Reviews and Meta-Analyses (PRISMA) (23) and its protocols (24). The PRISMA checklist is reported in Supplementary Table 1 of the Supplementary Materials.

Comprehensive searches were conducted in the PubMed, Web of Science, PsycINFO, and Google Scholar databases to identify relevant articles related to awareness and use of PEP among MSM. The search strings were intersections of disease-, population-, PEP-, and outcome-related terms (Supplementary Table 2 of Supplementary Materials). Additional searches were conducted in the Cochrane Library. To identify additional relevant citations, the reference lists of published reviews and journal articles were also screened. All searches were limited to English peer-reviewed journal articles. The original bibliographic searches for PsycINFO and PubMed were conducted on June 15, 2021. The original search for Web of Science, Google Scholar, and Cochrane Library were conducted on June 16, 2021. The updated searches for PsycINFO, PubMed, and Web of Science were conducted on November 19, 2021, while for Google Scholar and Cochrane Library on November 20, 2021.

### Selection Criteria

Studies were considered eligible if they met all of the following inclusion criteria: (1) conducted primary data collection; (2) were studies related to PEP use for HIV prevention; (3) reported data about gay and bisexual men, regardless of age, country, and HIV status; (4) reported history, awareness, or intention of PEP use among MSM; and (5) appeared in English-language, peer-reviewed journal articles. Studies that reported data about MSM and other populations, such as transgender people or sex workers, were included, but only data related to MSM were considered and abstracted. Studies were identified as ineligible if they were (1) case reports; (2) longitudinal studies or trend studies; (3) unpublished book chapters, theses, dissertations, or articles; and (4) non-original research, secondary reports, review articles, or theoretical framework articles.

### Study Selection, Data Abstraction, and Management

EndNote (version 6) was adopted throughout the process of study selection. First, titles and abstracts were preliminarily screened to exclude irrelevant studies. Second, full-text versions of selected papers were assessed independently to ensure that all inclusion criteria were met. Disagreement in the process of study selection was resolved by discussion with a third reviewer. The two researchers used standardized Microsoft Excel spreadsheets to extract the following information: authors, year of publication, country of study, study design, settings, study populations, outcomes, and factors. Interview quotes relating to awareness or use of PEP were also collected to aid conceptual understanding of qualitative findings.

### Data Analyses

The primary outcomes of interest were measures of PEP awareness and previous use or potential usage intention (Supplementary Table 3 in Supplementary Materials). All available data were pooled and synthesized using a combination of single-rate meta-analysis and a narrative synthesis approach. For quantitative studies, a random effects model was used to quantitatively summarize the pooled event rates (ERs) of PEP awareness and previous PEP use. Random effects meta-regression was used to assess the relationship between year of data collection and PEP awareness or use. Subgroup analysis was performed to determine the potential influence of the following covariate: level of national economic development.

A synthesis of factors determining PEP awareness and use was performed and classified into individual, social, or structural layers based on a socioecological model (25, 26). Qualitative data were drawn to provide context for the quantitative findings as recommended (27, 28) by identifying participant perspectives about factors that might affect awareness and previous use or potential intention of use of PEP among MSM. Thematic analysis was used to abstract, sort, compare, and categorize data to construct a set of emerging descriptive themes from relevant quotes. Themes were then used to construct a conceptual framework of awareness and previous use or potential usage intention of PEP in the individual, social, and structural domains.

### Bias Assessment

Two independent reviewers used the Cochrane Collaboration tool to assess the risk of different types of biases, including recruitment, sampling, attrition and non-response, social desirability, and researcher (29). The potential risk of impact of biases on the robustness of the included findings was also discussed (see Supplementary Table 4 in Supplementary Materials).

## Results

### Study Screened and Reviewed

**Figure 1:**
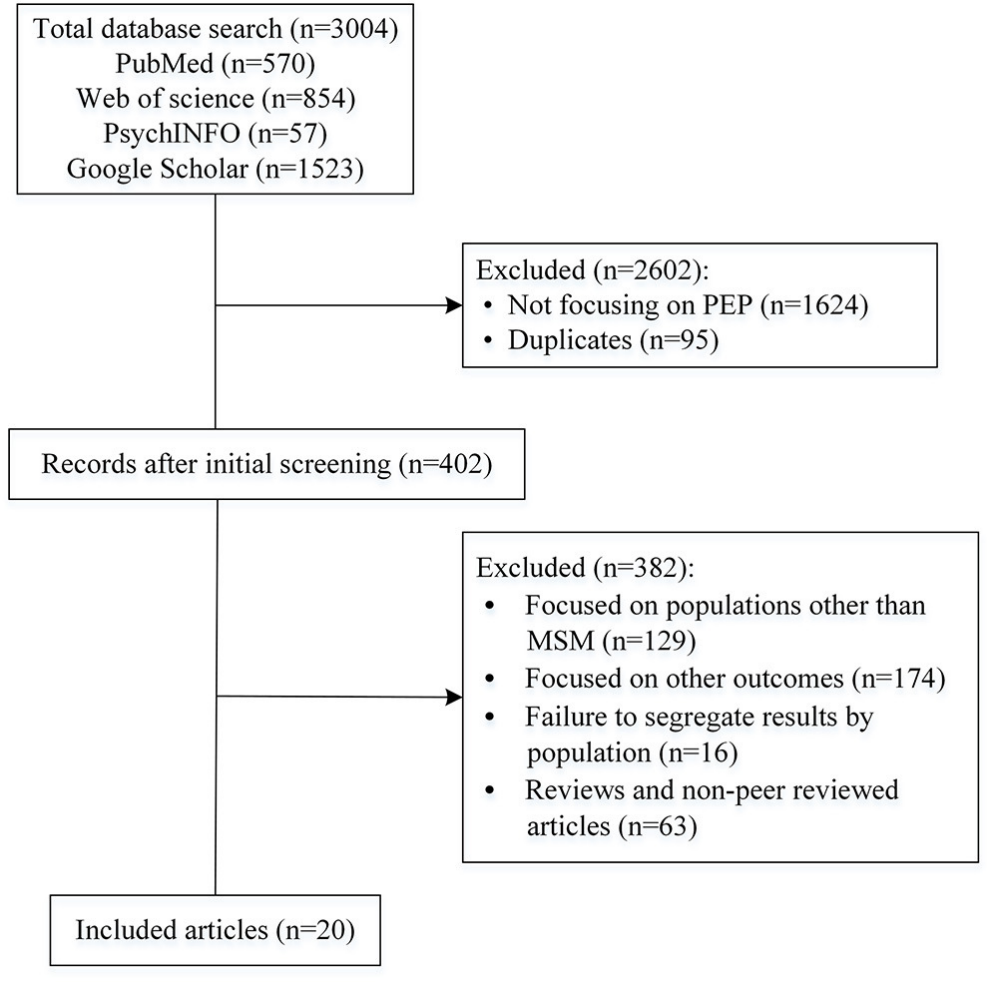
Flow chart of included studies.

Of a total of 3,004 titles, 402 were further assessed for inclusion, and 20 were included in the final review (Figure 1). The initial screening excluded studies that did not specifically focus on HIV, PEP, and duplicates, leaving a total of 402 citations. Subsequently, 382 citations were excluded after screening the abstracts and full papers. Several papers focusing on other population groups (e.g., heterosexual, sex workers, health care workers, and transgender populations) were identified through the search and were excluded (*n* = 129). Additional exclusions were due to poor relevance of outcomes (e.g., cost effectiveness, medication adherence; *n* = 174), failure to segregate results by population (*n* = 16), and reviews or non-peer reviewed articles (*n* = 63).

### Study Characteristics

Overall, 20 eligible studies published between 2007 and 2021 were included in the meta-analysis, involving 12,579 MSM. Supplementary Table 5 in the Supplementary Materials presents the key characteristics of the studies included in the review. Of the included studies, 19 were quantitative, and one was a mixed-method study. The two-phase mixed-method study (30) included a cross-sectional survey with a self-administered questionnaire and a prospective descriptive study using an in-depth interview, but data needed were collected in the quantitative survey phase. Another study used a longitudinal cohort study to evaluate the impact of HIV leadership programs, and its baseline survey provided the data we needed (31). All of the remaining quantitative studies were cross-sectional surveys.

### Description of PEP

The included studies used consistent definitions of PEP for MSM with different terms, such as non-occupational PEP (nPEP) or PEP after sexual exposure (PEPSE). Given that many persons did not know or knew little about PEP, an introductory explanation of PEP may have been provided to participants during the survey.

### Awareness of PEP

All 20 studies reporting awareness of PEP employed a simple binary question asking participants whether they were aware (or had heard) of PEP, except for one study (32) that asked whether a medical treatment existed that could reduce the chance of becoming HIV-positive.

**Figure 2:**
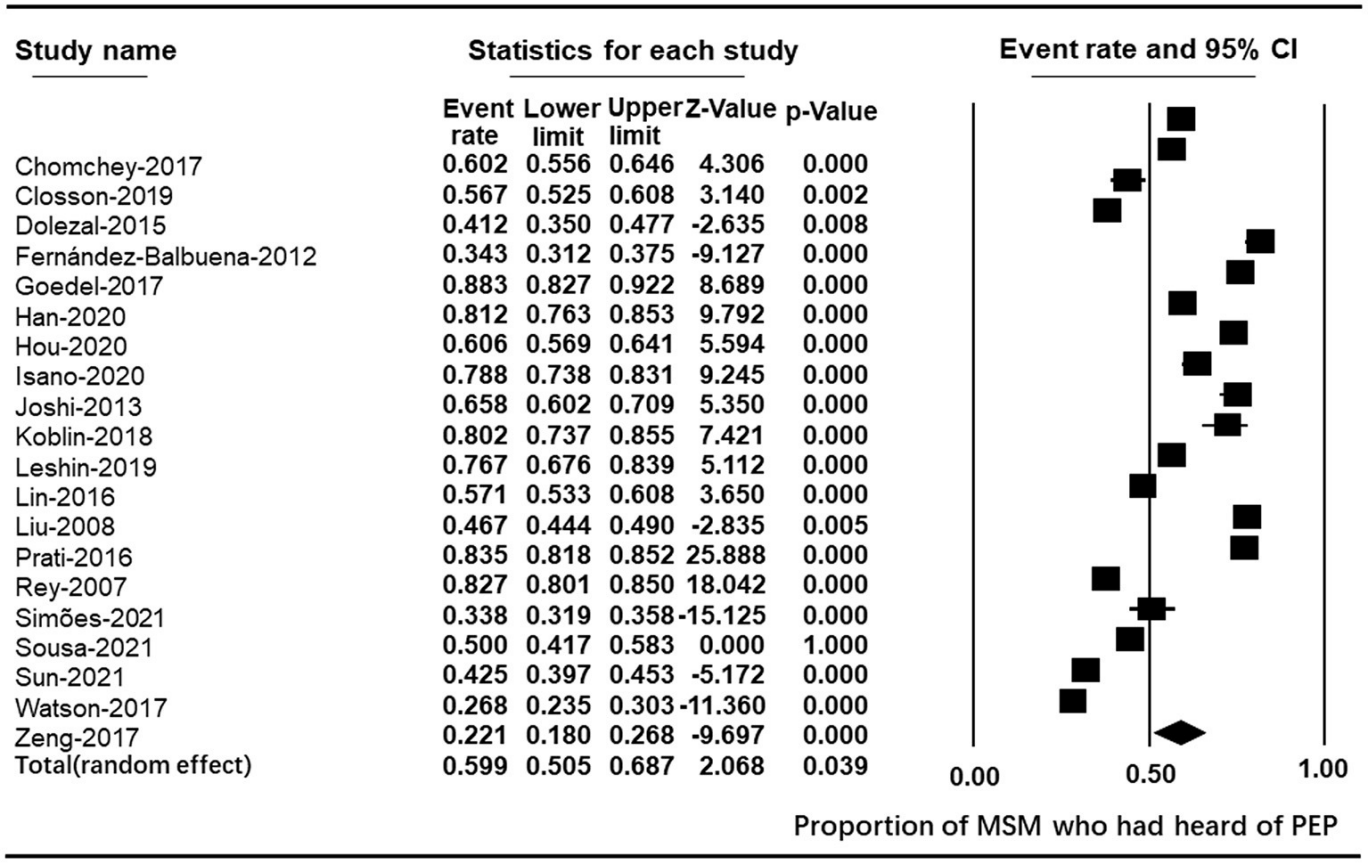
Forest plot of PEP awareness.

Meta-analysis of the 20 studies found that the pooled estimate of the proportions of MSM who were aware of PEP was 59.9% (95% CI: 50.5~68.7) (Figure 2). Awareness of PEP in these studies was inconsistent, ranging from 22.09% among MSM in Guangxi, China (33), to 88.27% among MSM in London who use geosocial networking smartphone applications (11). Seven studies reported higher awareness than 70% among gay men in Israel (15), young MSM of color in the United States (34), MSM users of geosocial networking applications in England (11), non-HIV-positive MSM in Italy (32), sexually active MSM living with HIV in France (35), MSM in 22 Sub-Saharan African countries (36), and among MSM college students in three cities of China (37). Four studies reported much lower levels of awareness (<40%) among MSM in China (33); MSM tested in community centers in Portugal (38) MSM receiving rapid HIV testing in Spain (39), and among black MSM in the United States (13). Nine studies reported modest PEP awareness: 41.23% among men engaging in condomless anal sex with men in the United States (8), 42.5% among MSM in Beijing, China (16), 46.67% among gay and bisexual men in California (40), 50% among MSM in Brazil (41), 56.70% among sexually active MSM in Vancouver, Canada (31), 57.06% among MSM in Vancouver, Canada (42), 60.22% among Thai MSM (30), 60.6% among MSM in four cities of China (43), and 65.77% among HIV-positive MSM in England (44). There was significant heterogeneity between studies (Q statistic = 1990.122, I^2^ = 99.045; *p* < 0.001).

Two studies equated awareness with knowledge of PEP (32, 40, 45). However, self-reported awareness did not necessarily reflect a precise understanding of PEP. Several studies checked whether self-reported knowledge about PEP was accurate or evaluated PEP knowledge in MSM. Those MSM who were aware of PEP had adequate knowledge about PEP; however, less than half of them correctly answered that PEP needs to be taken for 28 days (30, 34). Among 94 MSM who had heard of PEP, 11% were concerned about side effects; most (68%) felt they would know how to get PEP, and nearly two-thirds anticipated that they would not be able to afford it (8). Only 40% of those who reported being aware of PEP (24% of total participants) were aware that the time window for an effective PEP is 72 h (15).

### Factors Associated With PEP Awareness

Bivariate meta-regression showed that temporal changes could not explain PEP awareness (slope = −0.031, 95% CI:-0.038~-0.024; *p* < 0.001): the minimum value of PEP awareness was collected in 2016 (33), and the maximum value was also gleaned in 2016 (11). Subgroup analysis revealed that developed countries PEP awareness had no differences compared to that in developing countries (Q statistic = 0.865; *p* = 0.352): fourteen studies from developed countries found that the point estimate of proportions of MSM who were aware of PEP was 51.9% (95% CI: 50.9~53), and six studies from developing countries found the point estimate was 52.9% (95% CI: 51.1~54.7). In addition, the studies had no significant publication bias in the awareness subgroup analysis (intercept = 8.544, 95% CI:−2.2~19.29, *p* = 0.112).

In addition to overall awareness, seven studies explored other factors that were correlated with awareness of PEP among MSM. Table 1 illustrates the range of factors affecting MSM's awareness of PEP documented in the included studies. These factors, which could potentially impede or facilitate participant's awareness of PEP, were conceptually divided into different categories within the individual, social (including partners, families, and communities), and structural domains (health systems and legal factors). In the individual domain, low educational attainment, unemployment, having casual partners, and closeted bisexual were negative factors; high-level education, white race/ethnicity, gay sexual identity, knowing the HIV status of one's self, higher personal sexual altruism, metropolitan resident, higher annual income, using the internet as the main way of meeting partners, having sex with an HIV-positive male partner, and having unprotected anal sex were positive factors. How age, drug use, and number of partners exert influence on awareness of PEP is uncertain.

**Table 1:** Factors affecting awareness of HIV post-exposure prophylaxis (PEP) among men who have sex with men.

**Domains**	**Negative factors**	**References**	**Positive factors**	**References**
Individual factors	Low educational attainment	(35)	High-level education	(16, 32, 37–39, 41–43)
	Closeted bisexual	(13)	Knowing about HIV status of self	(11, 43)
	Using speed/crystal	(40)	White race/ethnicity	(40, 42)
	Unemployment	(35)	Gay sexual identity	(40, 42)
	Higher number of partners	(11)	Higher number of partners	(39, 42)
	Having casual partners	(35)	Higher personal sexual altruism	(42)
			Metropolitan resident	(8, 42)
			Greater perceived agency to ask sexual partners' HIV status	(42)
			Older age	(31, 35, 37, 40)
			Higher annual income	(16, 40, 43)
			Having unprotected anal sex	(40)
			Having sex under the influence of a drug	(37, 40, 41)
			Younger age	(16, 32)
			Using Internet as the main way of meeting partners	(39)
Social factors			Interaction with gay culture	(39)
			Internet and community of MSM	(33)
			Lower levels of HIV stigma	(16, 32)
			HIV leadership programming	(31)
Structural factors			Disclosure of one's sexual orientation to general practitioner	(11)
			Greater access to condoms	(42)
			Previous HIV testing	(11, 15, 32, 38, 43)
			Previous sexually transmitted infection (STI) diagnosis	(38, 42)
			Contact with HIV/AIDS organization	(32)

Social factors associated with greater awareness among MSM included more interaction with gay culture (39), lower levels of HIV stigma (32), and HIV leadership programming attendance (31). One study conducted logistic regressions predicting awareness of PEP and found that location was the only significant predictor: compared to Puerto Rican participants, those in Pittsburgh were 5.7 times more likely to have heard of PEP, and those in Boston were 10.1 times more likely to have heard of PEP (8).

Regarding structural factors, greater access to condoms (42), more frequent contact with HIV/AIDS organizations (32), previous sexually transmitted infection (STI) diagnosis (42), HIV testing (32, 34, 35, 43), and disclosure of one's sexual orientation to their general practitioner (11) were associated with having heard of PEP.

### Previous Use or Potential Intent to Use PEP

**Figure 3:**
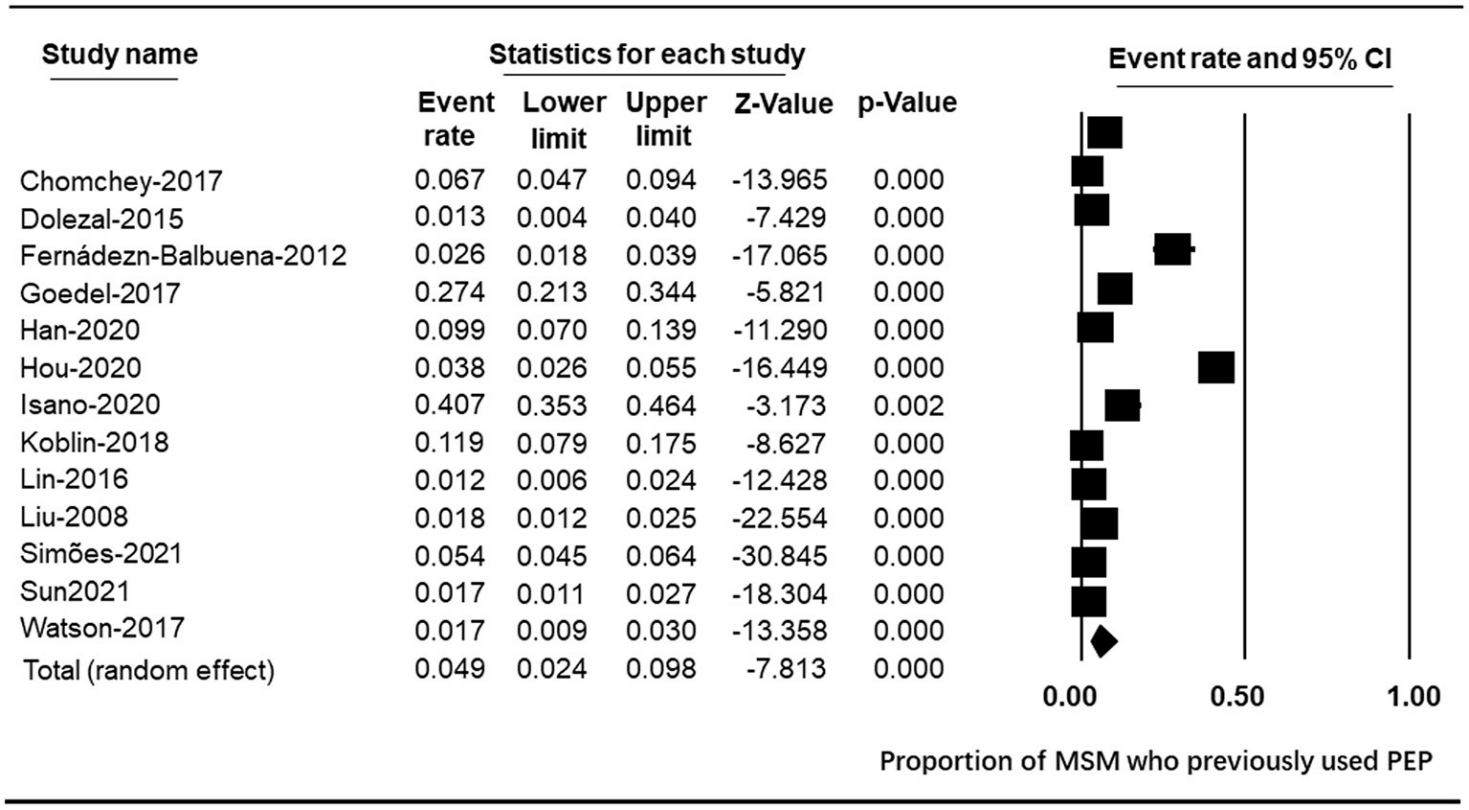
Forest plot of PEP use.

Thirteen studies assessed quantitative proportions of participants who had used PEP. All of them used a simple binary question, asking participants whether they had used or taken PEP in the past. Although the question was asked of all MSM participants in some studies (11, 13, 30, 39), we calculated the proportion of MSM who had used PEP among subsamples who were aware of PEP. Meta-analysis of the thirteen studies found that 4.9% (95% CI: 2.4~9.8) of MSM had used PEP (Figure 3). Previous use of PEP in these studies was low and inconsistent, ranging from 1.19% among HIV-negative MSM in Vancouver, Canada (42) to 40.74% among MSM in 22 Sub-Saharan African countries. Most studies reported much <10% of respondents that indicated previous use, with the exception of three studies that reported previous use of 40.74% among 22 Sub-Saharan African countries (36), 11.86% among young MSM of color in the United States (34), and 27.37% in London (11). As expected, there was a high level of heterogeneity between these studies (Q statistic = 656.703, *I*^2^ = 98.173, *p* < 0.001).

One study reported a qualitative assessment of willingness to use PEP, asking participants “If you have risk behaviors for HIV infection, will you seek PEP?,” and 416 participants (92.4%) answered yes (30). Another study (8) showed that participants were especially likely to say they would use PEP in the future, scoring an average of 9.1 (on a 10-point scale, with 10 being extremely likely), but did not report the proportion of MSM participants who were very willing to use PEP. One study used a single item with a 5-point Likert-type scale to assess willingness to use PEP and reported 461 (73.06%) MSM participants who were very-to-extremely likely to use PEP (45).

### Factors Associated With PEP Use

Bivariate meta-regression showed that years of data collection could predict PEP use (slope = −0.031, 95% CI:−0.038~-0.024; *p* < 0.001): PEP use among MSM significantly increased from 4.18% in 2006 to 30.01% in 2016 (11). Subgroup analysis revealed that PEP use in developing countries was significantly higher than that in developed countries (Q statistic = 96.77; *p* < 0.001): eight studies from developed countries found that the point estimate of proportions of MSM who previously used PEP was 5.4% (95% CI: 4.8~6.1), and five studies from developing countries found the point estimate was 13.2% (95% CI: 11.5~15). The studies had no significant publication bias in the use subgroup analysis (intercept = −7.260, 95% CI:−18.18~3.66, *p* = 0.171).

In addition to the overall previous use of PEP or willingness to use PEP, our review provides comprehensive information regarding barriers and facilitating factors of its use. Table 2 illustrates the range of factors influencing MSM's previous use or potential use of PEP mentioned in the eligible studies. Several studies explored factors that were associated with previous use of PEP among MSM. In the individual domain, concern about side effects (8) and lack of PEP information (11, 34) were barriers, whereas having adequate knowledge about PEP (30), having been involved in high-risk sexual intercourse (39), being circumcised (30), and using methamphetamine (11) were facilitating factors. Associations between past PEP use and club drug use have been tested for HIV in the past year, and recent sexually transmitted infection (STI) diagnoses have not persisted (11). In the social domain, being in a relationship (11) was a facilitating factor. In the structural domain, cost emerged as an important barrier to the use of PEP in some cities in the United States (8) or sometimes in Thailand, where PEP is not available free of charge (30).

**Table 2:** Factors affecting the use of HIV post-exposure prophylaxis (PEP) among men who have sex with men.

**Domains**	**Barriers**	**References**	**Facilitating factors**	**References**
Individual factors	Concern about side effects	(8)	Having been involved in high-risk sexual intercourse	(37, 39)
	Lack of PEP information	(11, 34)	Using methamphetamine	(11)
			Having an adequate knowledge about PEP Knowledge where to get PEP	(30, 36, 43) (36)
			Being circumcised	(30)
Social factors	Being refused housing Experiencing abusive language	(36) (36)	Being in a relationship	(11)
Structural factors	Expense of PEP	(8, 36)		

Factors associated with an intention to take PEP included awareness of PEP, HIV knowledge with > 80% correct answers, absence of penetrative anal sex in the past 3 months, and circumcision (30). Partnered men's willingness to use PEP was positively associated with having an individual income < $30,000 USD and serosorting within the relationship (sexual partner selection on the basis of HIV status), whereas it was not clear why higher investment in couple relationships and greater age differences between primary partners were barriers (45).

## Discussion

The finding of modest PEP awareness may be because high-income countries possess an apparent advantage with coverage of antiretroviral therapy, HIV diagnosis among people living with HIV, and access to health care services (3). MSM, as a key population for HIV prevention, have recently received a great deal of attention, especially in cities of high-income countries. For example, a trend study in Australia found that awareness of PEP among gay men significantly increased from 23% in 2001 to 64% in 2010 (46). In Europe, researchers saw an opportunity to provide global leadership at the regional scale-up of comprehensive AIDS prevention interventions for MSM (47).

In many low- and middle-income countries, post-exposure prophylaxis is not yet a routine service, and there is relatively little research on it. This review lacks data from low- and middle-income settings. Most included studies (8/20) were conducted in the United States, England, and Canada, which limited the extent to which the findings may be applicable to MSM in low- and middle-income countries because factors that drive trends in HIV prevention among MSM may be very different among them. On the one hand, a body of evidence reports that MSM are at marked risk for HIV infection in low- and middle-income countries in Asia, Africa, Latin America, and Eastern Europe (48, 49). On the other hand, MSM are a markedly underserved and underresourced population in low- and middle-income settings such as Latin America (50) and Africa (51). There is an urgent need to better understand awareness and use of PEP, as well as to improve HIV prevention among MSM in low- and middle-income countries.

Appropriate use of PEP is extremely low compared with HIV prevalence and high-risk behavior among MSM. After high-risk exposure to HIV, very few MSM take PEP. A study found that only three had used PEP among 228 men engaging in condomless anal sex with men (8). Even if some MSM had used or were willing to use PEP, they may not adhere to completing the PEP regimen. A meta-analysis revealed that adherence to the full 28-day course of antiretroviral drugs prescribed for PEP was only 67.2% (17). In some countries, PEP services often depend on emergency physicians and specialists (IDs) in HIV/sexually transmitted infection clinics, which can potentially hinder the use of PEP (52, 53). Recently, one study showed that the mean knowledge scores were low for health care workers in campus clinics and tertiary hospitals (54). Some strategies are required to improve the appropriate use of PEP for HIV prevention among MSM when at risk. PrEP and PEP are highly validated HIV prevention tools and are part of a wide range of prevention measures, including HIV testing, condom use, and screening and treatment for sexually transmitted infections (55). We should train community physicians in risk assessment, medication adherence assessment, and offline communication through online social media and offline posters, cards and brochures to promote PEP knowledge and inform them of ways to seek services (56). The completion and use of PEP has been shown to vary depending on the drug regimen. In many countries, tenofovir disoproxil fumarate (TDF)/emtricitabine (FTC) is the preferred backbone drug (57, 58). However, in the MSM population, the vast majority of MSM prefer to use a single dose of PEP as opposed to a multitablet regimen formulation, and single-dose regimens show better adherence (59, 60). An optimized regimen with good tolerability and simplified use improves PEP adherence. We only conducted subgroup analysis of very limited geographic factors, which is one of the limitations of this review. MSM included in our meta-analysis are diverse, with different ages, educational attainment, races/ethnicities, annual incomes, sexual identities, HIV statuses, and so on. Sociodemographic comparisons could provide further insights into contextual determinants of awareness or use of PEP. As mentioned earlier, the reported proportions of PEP awareness and use were highly heterogeneous. The diversity of recruitment methods and data collection settings may have also influenced the precision of our estimates, and other factors, such as differences in MSM participants and their settings, could also contribute to bias. Therefore, we used the random effects model rather than the fixed effects model, as the former provides more conservative estimates, such as wider 95% confidence intervals (CIs).

This review provides information regarding factors associated with PEP awareness, which is crucial for the promotion of treatment-seeking behavior after sexual exposure and reduction of the HIV transmission rate among MSM. In particular, this review examined a series of individual, social, and structural factors that may influence this awareness. These findings about individual factors suggest that PEP education and information should be prioritized for MSM if they have a low educational level, are unemployed, have casual partners, are ethnic minorities, and are closeted bisexual men. In the social domain, lower levels of HIV stigma and more interaction with gay culture were positively associated with higher awareness, but other factors were not investigated sufficiently, such as stigmatization of PEP and homosexual orientation and reaction or support from partners, peers, and family. In the structural domain, access to condoms, HIV testing, STI diagnosis, and primary providers or general practitioners having accurate knowledge about MSM behavior and sexual orientation could play important roles in awareness of PEP. Stigma from peers, partners, and family as well as health care providers continues to be perceived by MSM (61, 62), and criminalization of same-sex relations may limit uptake of prevention services among MSM (1), so it is essential to integrate strategies to mitigate stigma related to sexual orientation and create an MSM-friendly environment, in addition to providing PEP information and education.

Our review also provides timely and comprehensive information regarding motivations for and barriers to PEP use, but many factors have not been fully discussed. Although PEP is effective in reducing HIV transmission, it may also exacerbate high-risk sexual behavior. The relationship between PEP use and high-risk sexual behaviors is unclear. PEP may exacerbate high-risk sexual behaviors, also known as sexual risk behavior disinhibition PEP (15, 63). However, some studies have shown no significant correlation between the two (64–66). In this regard, it is necessary to conduct research in the future to evaluate the potential impact of PEP on the sexual behavior of key populations. Except for concern about side effects, lack of PEP information, and high-risk behaviors, several other individual-based demographic factors, including levels of educational attainment, age, race/ethnicity, residency, and sexual orientation, could influence PEP use but were not well researched. Individual behaviors of MSM, such as engaging in condomless sex, are often shaped in social contexts. In addition to the relationship and cost of PEP, other key social and structural factors, including reaction or support from relatives and friends, stigmatization in health facilities, concern for quality of treatment, and a lack of confidentiality and privacy protection, should be further explored. As MSM increasingly turn to the internet to find the community and meet partners, internet-based platforms are becoming salient social environments for MSM and could offer opportunities for HIV prevention (67).

Notwithstanding several limitations, our review points to several considerations for future PEP research, policy, and clinical practice. (1) To better understand global AIDS prevention, awareness and use of PEP among MSM in low- and middle-income countries and areas should be surveyed. (2) A fuller understanding of the reasons for PEP awareness and use among MSM is needed. (3) To make significant progress in AIDS prevention and control, the barriers found in our review and other potential barriers should be addressed.

Post-exposure prophylaxis is important not only to reduce HIV transmission in MSM, but also in other high-risk populations. Examples include heterosexual populations, injection drug users, bisexual populations, and sex workers (43, 68). Some studies show that MSM report higher awareness of PEP than other at-risk groups for HIV (12). A study in Brazil found that MSM participants were more likely to have knowledge about PEP than heterosexual male participants (41). Awareness of PEP may be associated with the age, higher education, and income of these MSM in the high-risk population. In conclusion, PEP has been shown to be safe and efficacious in reducing the risk of HIV acquisition (69, 70), and demonstration projects are becoming increasingly being implemented (71–73). This review reveals that awareness of PEP among MSM is generally modest, ranging from 22.09 to 88.27%. However, PEP is underused, with a pooled estimate of 4.3% among MSM. Despite the currently low previous use of PEP, this review contributes to this evidence base by demonstrating that MSM are willing to use PEP when they become aware of it and that they should obtain various sources of support to cope with all kinds of barriers mentioned in this article. Programs aimed at introducing or promoting the usage of PEP need to be based on context-specific evidence, such as potential demand and user preferences, and need to be supported by enabling policies and legal framework environments.

## Transparency Declaration

BS affirms that this article is an honest, accurate, and transparent account of the study being reported; that no important aspects of the study have been omitted; and that any discrepancies from the study as planned (and, if relevant, registered) have been explained.

## Data Availability Statement

The original contributions presented in the study are included in the article/Supplementary Materials, further inquiries can be directed to the corresponding authors.

## Author Contributions

RS, HZ, and BS conceptualized the study. JJ, RS, TM, and TJ searched the literature, selected studies, and extracted the data. JJ, RS, TM, LD, HL, XR, JC, JY, LS, HW, TZ, and HZ contributed to the analysis, interpretation of the data, and provided important scientific input. JJ, RS, and BS analyzed the findings and wrote the first draft of the manuscript. BS supervised the whole study. All authors collaboratively discussed key decisions throughout the course of the review, provided critical feedback on preliminary manuscript and interpretation of results, and approved the final version.

## Funding

This work was supported by the National Natural Science Foundation of China (NSFC, 81772165 and 81974303 to BS, 82072271 to TZ, 81703278 to HZ), the NSFC-NIH Biomedical collaborative research program (81761128001 to HW), the Climbing the peak (Dengfeng) Talent Training Program of Beijing Hospitals Authority (DFL20191701 to TZ), Special Project of Scientific Research and Cultivation of Beijing Centers for Disease Prevention and Control/Center for Preventive Medicine Research (2020-BJYJ-14 to XR), and Beijing Key Laboratory for HIV/AIDS Research (BZ0089). The funders had no role in study design, data collection and analysis, decision to publish, or preparation of the manuscript.

## Conflict of Interest

The authors declare that the research was conducted in the absence of any commercial or financial relationships that could be construed as a potential conflict of interest.

## Publisher's Note

All claims expressed in this article are solely those of the authors and do not necessarily represent those of their affiliated organizations, or those of the publisher, the editors and the reviewers. Any product that may be evaluated in this article, or claim that may be made by its manufacturer, is not guaranteed or endorsed by the publisher.

## Abbreviations

PEP: post-exposure prophylaxis
PrEP: pre-exposure prophylaxis
nPEP: non-occupational PEP
PEPSE: PEP after sexual exposure
MSM: Men who have sex with men
HIV: Human immunodeficiency virus
AIDS: acquired immunodeficiency syndrome
PROSPERO: Prospective Register of Systematic Reviews
PRISMA: Preferred Reporting Items for Systematic Reviews and Meta-Analyses
ER: event rates
STD: sexually transmitted disease
STI: sexually transmitted infection
CIs: confidence intervals.
