# Supplementary material for: Awareness and Use of Post-exposure Prophylaxis for HIV Prevention Among Men Who Have Sex With Men: A Systematic Review and Meta-Analysis
Source: Front Med (Lausanne). 2022 Jan 10;8:783626. doi: 10.3389/fmed.2021.783626 (PMC8784556; doi:10.3389/fmed.2021.783626)
Supplement: Supplementary file 2 [file Table_2.DOCX]

**Table S2. Items included in the comprehensive database search**

| HIV/AIDS | PEP | MSM | Awareness | Use |
| --- | --- | --- | --- | --- |
| HIV | Post-exposure prophylaxis | Men who have sex with men | Awareness | Intention to use |
| AIDS | PEP | MSM | Knowledge | Willingness to use |
| Human immunodeficiency virus | Non-occupational post-exposure prophylaxis | Gay | Know | Uptake |
| Acquired immune deficiency syndrome |  | Bisexual men |  | Use/usage |
