# Supplementary material for: Awareness and Use of Post-exposure Prophylaxis for HIV Prevention Among Men Who Have Sex With Men: A Systematic Review and Meta-Analysis
Source: Front Med (Lausanne). 2022 Jan 10;8:783626. doi: 10.3389/fmed.2021.783626 (PMC8784556; doi:10.3389/fmed.2021.783626)
Supplement: Supplementary file 3 [file Table_3.DOCX]

**Table S3. Primary and secondary outcomes of this review**

| **Outcome level** | **Definition** |
| --- | --- |
| Awareness of PEP | Proportion of MSM participants who reported knowing about PEP |
| History of PEP use | Proportion of MSM participants who reported having used PEP in the past |
| Intention of PEP use | Proportion of MSM participants who reported a commitment or willingness to use PEP for HIV prevention following risk exposure |
| Factors affecting awareness of PEP | Individual, social, or structural factors that may determine the awareness of PEP |
| Factors affecting PEP use | Individual, social, or structural factors that may determine the history or intention of PEP use |
