# Supplementary material for: Awareness and Use of Post-exposure Prophylaxis for HIV Prevention Among Men Who Have Sex With Men: A Systematic Review and Meta-Analysis
Source: Front Med (Lausanne). 2022 Jan 10;8:783626. doi: 10.3389/fmed.2021.783626 (PMC8784556; doi:10.3389/fmed.2021.783626)
Supplement: Supplementary file 4 [file Table_4.DOCX]

**Table S4. Source of Bias.**

| **Source of Bias** | |
| --- | --- |
| Recruitment | One study recruited a convenience sample of MSM exclusively through online methods and used only one Internet-based tool, that is, a popular geosocial-networking smart phone app (Goedel et al., 2017), which excluded those who did not have access to the app. MSM who used the app to meet sexual partners may have more interaction with gay culture, and therefore were more likely to heard of PEP. Two studies used Internet-based advertisement and recruitment as well as newspaper advertisements (Dolezal et al., 2015) or flyers distributed in public spaces that served the gay community (Watson et al., 2018).  Nine studies exclusively used face-to-face recruitment to investigate MSM who were being tested for HIV (Chomchey et al., 2017; Fernandez-Balbuena et al., 2013; Leshin et al., 2019), receiving HIV care (Joshi et al., 2014; Rey et al., 2007; Zeng et al., 2017), or appearing at venues such as bathhouses, bars, pool parties, dance events, and so on (Liu, 2008; Hou et al., 2020). This approach may have excluded MSM who did not have contact with HIV services or MSM-related venues. For example, Leshin et al. (2019); Simões et al. (2021) evaluated awareness of PEP among a large cohort of individuals tested for HIV following unprotected sexual intercourse, neglecting MSM who didn’t seek for HIV test after high-risk sexual behavior.  Seven studies used face-to-face recruitment or in-person recruitment at various events in combination with online methods, including local agencies (Lin et al., 2016; Isano et al., 2020; Han et al., 2020; Sousa et al., 2021; Sun et al., 2021), clinics, bars, clubs, and other social scenarios, (Dolezal et al., 2015; Koblin et al., 2018). These events were thought to be frequented by high-risk MSM, but these strategies might have excluded people who were not living openly as MSM.  In addition, recruitment from rural areas and low- and middle-income countries across all studies was limited. Six studies were conducted in the United States, two studies were conducted in England, and two in Canada. Spain, sub-Saharan Africa, Brazil, Portugal ,Italy, France, Thailand, and China were also included. |
| Sampling | Most studies used convenience sampling; the sampling method used by other studies included time-venue sampling (Liu, 2008), stratified random sampling (Rey et al., 2007), peer-recruited sampling, or respondent-driven sampling (Lin et al., 2016). These sampling methods all had some inherent biases.  One study recruited sexually active MSM into a longitudinal cohort study using respondent-driven sampling (RDS) (Closson et al., 2019). In RDS, the structure of the social contacts defines the sampling process and affects its coverage, for instance by constraining the sampling within a sub-region of the network. |
| Attrition and nonresponse | Five studies reported instances of nonresponses, incompletely filled questionnaires, or missing data (Dolezal et al., 2015; Koblin et al., 2018; Lin et al., 2016; Liu, 2008; Prati et al., 2016), which introduced response bias and may limit generalizability. One study reported that 44% of eligible participants did not take part due to time constraints (Chomchey et al., 2017). |
| Social desirability | Social desirability may seriously bias data both in experimental research and in surveys. The potential for social desirability bias may have been accentuated in studies that involved face-to-face standardized questionnaires administered by a trained interviewer (Liu, 2008; Rey et al., 2007; Sun et al., 2021) and particularly mitigated by the use of anonymous online data collection methods in four studies (Goedel et al., 2017; Prati et al., 2016; Han et al., 2020 ;Isano et al., 2020). Four studies used a computer-assisted self-interview (Closson et al., 2019; Dolezal et al., 2015; Lin et al., 2016; Watson et al., 2018), which may improve the self-report of stigmatized behaviors. |
| Researcher | Studies using face-to-face surveys contained limited documentation of survey dynamics, interactions, emotions, or beliefs of the researchers, especially when discussing sensitive topics. The absence of this information may have limited our analysis of the researchers’ influence on the research conduct and reported findings. |
