# Supplementary material for: Awareness and Use of Post-exposure Prophylaxis for HIV Prevention Among Men Who Have Sex With Men: A Systematic Review and Meta-Analysis
Source: Front Med (Lausanne). 2022 Jan 10;8:783626. doi: 10.3389/fmed.2021.783626 (PMC8784556; doi:10.3389/fmed.2021.783626)
Supplement: Supplementary file 5 [file Table_5.DOCX]

**Table S5. Characteristics and findings of studies included in the systematic review**

| **Study** | **Country and setting** | **Design** | **Sample size** | **Participants’ characteristics** | **Awareness** | **Had used** | **Intention** | **Factors related to PEP awareness and use** |
| --- | --- | --- | --- | --- | --- | --- | --- | --- |
| Chomchey et al., 2017 | Bangkok, Thailand | Mixed method (survey and interview) | 450 MSM | Mean age = 26.8 (6.1), 67.3% had a Bachelor’s degree or higher, 46.7% were employed, 18.9% were HIV-positive | 271 (60.2%) aware | 30 (6.7%) had experience of nPEP | 416 (92.44%) intended to seek nPEP if at risk. | Factors associated with an intention to take nPEP were awareness about nPEP, HIV knowledge, mode of sexual intercourse, and circumcision. |
| Closson et al., 2019 | Vancouver, Canada | Survey  (online) | 552 MSM | 36.8% were less than 30 years old at first visit, 93.4% completed high school, 75.5% were White, 71.19% were HIV-negative. | 313(56.70%) had heard of PEP. |  |  | Participants who have heard of PEP were less likely to be young, and more likely to report ever participating in a leadership program. |
| Dolezal et al., 2015 | Boston, Pittsburgh, and San Juan, United States | Survey  (online and face-to-face) | 228 MSM engaging in condomless anal sex | Mean age = 23.1 (3.3), age range = 18–30 years, annual income was 14,249 USD on average, 41% were white, 44% were Latino, 80% were gay, 61% were employed, 26% had an STD, including HIV. | Totally, 94 (41.23%) had heard of PEP. Whereas 64% of Boston participants and 47% of Pittsburgh participants knew of PEP, only 16% of those in San Juan knew of it. | 3 (1.3%) had used PEP in the past. | Participants were especially likely to say they would use PEP in the future, scoring an average of 9.1 (on a 10-point scale, with 10 being extremely likely). | Among MSM who had heard of PEP, 11% would not use PEP due to concern about side effects, 68% did not know how to get it, 62% could not afford. |
| Fernández-Balbuena et al., 2012 | Mainly in the city of Madrid, Spain | Survey  (face-to-face) | 2545 HIV-negative men and women, including 870 MSM | 51.4% were aged 30 and over, 55.5% had a university degree, 83% had paid work, 74.5% were born in Spain, 78.5% were gay, 64.4% were frequenter of the gay scene, 25.4% never had HIV test. | 298 (34.25%) had heard of nPEP. | 23 (2.6%) had used nPEP. |  | MSM were less likely to be aware of nPEP if they had been born in Latin America, and more likely to be aware of nPEP if they had a university degree, were members of a gay community-based organization, or had used the Internet as their main way of meeting sexual partners over the past 12 months. Awareness increased with the number of sexual partners in the past 12 months and the number of previous HIV tests. |
| Goedel et al., 2017 | London, UK | Survey  (online) | 179 self-reported HIV-uninfected MSM users of an app. | 42.5% were aged between 18 and 30 years, 97.8% described themselves as gay or bisexual, 72% were white, 87.1% were currently employed or enrolled as a student, 22.3% were depressive, 39.7% had used alcohol in past 3 months, 78.8% had disclosed sexual orientation. | 158 (88.27%) reported ever hearing about PEP. | 49 (27.4%) reported having ever used PEP. |  | The disclosure of one’s sexual orientation to their general practitioner and reporting one’s HIV status as negative (rather than unknown) were associated with having heard of PEP. Recent methamphetamine use and currently being in a relationship with another man were associated with PEP use. |
| Hou et al., 2020 | Beijing, Changsha, Guangzhou,  and Shanghai  China | Survey  (online and in person) | 708 MSM | 69.6% had a college or above education, 70.6% were employed, 67.9% were gay, 29.8 were bisexual. | 429(60.59%) had heard of PEP. | 27(3.8%) had PEP use | 69.9% reported intended to  use PEP in the event of a potential exposure | Men with higher education, more frequent HIV testing, HIV seroconversions within their social network, and who only had sex with men. worry about becoming infected with HIV were significantly associated with PEP willingness and use |
| Han et al., 2020 | Beijing, Shenzhen and Kunming  China | Survey  (online) | 293 MSM who completed questionnaire effectively | 38.6% were aged 22-24 years, 91.1%were undergraduates., 64.5% had no income. | 238(81.2%) had heard of PEP. | 29(9.9%) had used PEP. | 62(21.2%) intended to seek nPEP | Higher education, more frequent HIV testing, sustaining condom use during sexual intercourse in recent 3 months, more than 1 male partner , having group sex, using rush, having sex with HIV-infected men were significantly associated with PEP awareness and use. |
| Isano et al., 2020 | sub-Saharan Africa  (SSA) | Survey  (online) | 29(including  Gay Bisexual  207MSM) | Mean age = 35.9years,70.1%  were single ,38.6% had achieved only secondary school education, 32.8% had attained University-level education,74.6% were gay, 19.3%were bisexual. | 234 (78.8%) had heard of PEP. | 121 (40.7%) had used PEP. |  | Eight factors were found associated with PEP use: educational level, having heard of PEP、Knowledge about where to get PEP、PEP availability at the health facility comfortable to discuss sexuality  with health care provider、being refused housing、experiencing abusive language or insults at the church or place of worship |
| Joshi et al., 2013 | London, UK | Survey  (face-to-face) | 298 HIV-positive MSM and 530 HIV-positive heterosexuals | 86.7% were aged 35 or older, 65.6% were diagnosed HIV-positive between 1985 and 2005. | 196 (65.77%) MSM were aware of PEP. |  |  | Overall, PEP awareness was unexpectedly low. MSM, younger patients (aged 19–34), and those diagnosed after 2006 were significantly more likely to be PEP aware. |
| Koblin et al., 2018 | New York, USA | Survey  (online and in person) | 177 HIV-uninfected young MSM of color (YMSMOC) | Aged 16–24 years, 75.3% identified as gay, 57.62% were black/African-American, 15.82% had less than high school education, 59.89% were employed. | 142 (80.23%) had heard of PEP. | 21 (11.9%) had used PEP. |  | Among YMSMOC, PEP awareness was significantly higher among those who were not employed and had an HIV test in the past 6 months. PEP awareness was significantly lower for those with 4 or more partners in the past 6 months. The mean score for potential barriers to PEP use was significantly lower for those aware of PEP than for those not aware. |
| Liu et al., 2008 | San Francisco Bay Area, Palm Springs, or San Diego, California, USA | Survey  (face-to-face) | 1819 HIV uninfected gay/bisexual men | Median age = 34 years, 60.39% identified as white, 89.82% identified as gay. | 849 (47%) reported PEP awareness. | 32 (1.8%) of 1819  respondents reported prior PEP use. |  | Men who were older than 25 years of age, were white, had an annual income of $100,000, self-identified as gay, and had unprotected anal sex or sex under the influence of a drug were more likely to be aware of PEP, whereas speed/crystal users were less likely to be aware of PEP. |
| Lin et al., 2016 | Vancouver, Canada | Survey  (online) | 524 HIV-negative/unknown MSM and 195 HIV-positive MSM | Median age = 33 years, 68.3% identified as white, 80.3% identified as gay, 76.7% were HIV-negative, 51.8% lived in downtown city, 65.7% had education levels higher than high school. | Of 673 participants who answered questions regarding nPEP awareness, 57.1% had heard of nPEP, including 272 (64.74%) HIV-negative/  unknown MSM and 112 (54.40%) HIV-positive MSM. | Only eight HIV-negative participants (1.2%) had previously used nPEP |  | Generally, nPEP awareness was higher for participants who engaged in sexual activities with high risk. Factors associated with greater awareness among HIV-negative participants included recent alcohol use, higher communal sexual altruism, previous sexually transmitted infection (STI) diagnosis, greater perceived condom use self-efficacy, white race/ethnicity, gay sexual identity, more formal education, higher personal sexual altruism, and Vancouver residence. Greater nPEP awareness among HIV-positive participants was associated with greater perceived agency to ask sexual partners’ HIV status and more frequently reporting doing so, a higher number of lifetime receptive sex partners, and greater access to condoms. |
| Leshin et al., 2019 | Jerusalem, Israel | Survey  (face-to-face) | 103 homosexual | Aged 18-35 years, almost. | 79 (76.70%) homosexual were aware of PEP. |  |  | Participants not aware of PEP featured a longer period from exposure to a decision to perform HIV test. |
| Prati et al., 2016 | Italy | Survey  (online) | 1874 non–HIV positive MSM | 60.3% were aged 30 and older, 68.6% had a college-level education, 51.8% were in a relationship. | 1543 (83.5%) had knowledge about PEP. |  |  | The variables most consistently associated with PEP awareness were contact with HIV/AIDS organizations, HIV testing, and HIV stigma. |
| Rey et al., 2007 | France | Survey  (face-to-face) | 2280 sexually active people living with HIV, including 930 gays | Mean age = 41 years | 769 (82.68%) homosexual were aware of PEP. |  |  | After multiple adjustment, factors associated with lack of PEP awareness were a low educational level, unemployment, older age, and CD4 cell counts <200. Individuals who reported having unprotected sex with a non–HIV positive steady partner also independently showed lower levels of PEP awareness. Reporting having casual partners was associated with better awareness. |
| Sun et al., 2021 | CDC  China | Survey  (online) | 1202 MSM | Mean age = 39.4years,  42.0% had a college degree or higher, 65.1% were homosexual orientation. | 511 (42.5%) had heard of PEP. | 21 (1.7%) had used PEP. |  | MSM with younger age,; a high level of education; and high income were more likely to be aware of nPEP. |
| Simões et al., 2021 | Portugal | Survey  (online) | 2275 MSM | The total number completed  questionnaires of people responding to at least one question of the PEP (n=12893), between January 2016 and December 2019 | 770 (33.8%) had heard of PEP. | 122 (5.4%) had used PEP. |  | PEP awareness in this group was associated with younger ages, higher education, and previous  HIV test. |
| Sousa et al., 2021 | Brazil | interview | 397participansIncluding 136 MSM | 21.9% were aged 25-34years, 37% were aged 35-44ears, 65.2% were employed | 68 (50%) had heard of PEP. |  |  | Who used alcohol during intercourse , the level of education, have a low viral load or did not know about their viral load were associated with PEP awareness and use. |
| Watson et al., 2017 | Atlanta, Georgia, USA | Survey  (online) | 650 black MSM reported being HIV-negative or of unknown  status | Mean age = 33.78 years, 54.12% had a college or above education, 70.6% were employed, 15.23% were HIV-positive. 46.74% were gay, 39.91 were bisexual. | 174 (26.8%) knew about PEP. | 11 (1.7 %) had used PEP. |  | Overall, the closeted bisexual class was the least likely to know about PEP compared to the managing minorities and gay, out, and open classes. |
| Zeng et al., 2017 | Guangxi, China | Survey  (face-to-face) | 344 MSM who completed questionnaire effectively | 77.6% were aged 21-40 years, 61.3% were Han Chinese, 34.9% were Zhuang nationality, 55.2% had education levels higher than junior college, 66.6% had low income. | 76 (22.1%) had heard of PEP. |  |  | They got information of PEP mainly by Internet and community of MSM. |
